# Assessing Essential New-Born Care Knowledge, Skills and Associated Factors among Nurses/Midwives in Zanzibar: A Cross-Sectional Study

**DOI:** 10.24248/eahrj.v7i1.709

**Published:** 2023-07-12

**Authors:** Salama A. Bakar, Angelina A Joho

**Affiliations:** a Department of Clinical Nursing, School of Nursing and Public Health, The University of Dodoma, Dodoma, Tanzania

## Abstract

**Background:**

Essential newborn care (ENC) is one of the significant strategies for neonatal survival, especially immediately after delivery. Nurses and midwives are the key healthcare providers who care for neonates immediately after birth, their knowledge and skills on ENC are very important for the preventable causes of neonatal deaths. Therefore, this study aimed to assess essential newborn care knowledge and skills among nurses/midwives in Zanzibar

**Methods:**

A hospital-based analytical cross-sectional study that included 246 nurses-midwives was conducted in Zanzibar from January to February 2021. The purposive sampling method was used to select district and regional hospitals. Simple random sampling was used to select primary health facilities. A systematic random sampling technique was used to select study participants. A standard structured self-administered questionnaire was used. Predictors of knowledge and skills of ENC were determined using Binary Logistic regression under multivariate analysis using SPSS version 23.0. P<.05 was considered to be significant.

**Result:**

Among the total (246) participants, 89 (36.2%) and 66 (26.8%) had adequate knowledge and appropriate skills of ENC, respectively. Having a BSc in Nursing (AOR = 8.83, 95%CI = 2.00-38.96) and the presence of guidelines (AOR = 3.52, 95%CI = 1.59 -7.80) were significantly associated with knowledge of ENC. Residing in Pemba (AOR = 0.30, 95%CI = 0.11-0.80), availability of staff (AOR = 0.80, 95%CI = 0.02-0.32), adequate knowledge (AOR = 2.80, 95%CI = 1.15-6.84) were factors significantly associated with ENC skills.

**Conclusion:**

Generally, nurses-midwives had suboptimal knowledge and skills on essential newborn care. Nurses-midwives are in urgent need of positive supportive supervision and low-dose– high-frequency skills training in ENC for the prevention of neonatal morbidity and mortality. Also, policymakers should be aware of this gap and should plan necessary interventions to close the gap to resecure newborns' survival.

## BACKGROUND

Globally, new-born deaths were estimated to reach 2.5 million in 2018. From these, nearly 7,000 new-born deaths were documented per day.^1^ Among these, 99% of the deaths occur in low-income nations and occur in the early stages after birth.^1^ In Sub-Saharan Africa, the neonatal mortality rate was approximately 28/1000 live births.^1^ In East African countries, the neonatal mortality rate ranges between 42 and 49 neonatal death per 1000 live births.^2^ In Tanzania, from 2010 to 2015, neonatal and infant mortality rates were between 25 and 43 deaths per 1000 live births, respectively.^3^

Essential new-born care is one of the significant strategies recommended by the WHO to promote the well-being of neonatal and prevent preventable neonatal deaths which usually happen within the first few days of life after birth.^6^ WHO delineates Essential New-born Care (ENC) as an all-inclusive strategy developed to strengthen the new-borns health by making interventions before pregnancy, during pregnancy, soon after birth, and during postnatal.^5^ These interventions, as recommended by WHO are crucial for all newborns irrespective of their socio-demographic factors ^1^

The availability of skilled nurses and/or midwives to provide ENC prevents 75% of new-born deaths during delivery and postnatal period, respectively.^5^ However, three-fourths of nurses/ midwives do not have the necessary skills to provide ENC as documented in a study conducted in Nigeria.^6^ This finding is against the WHO's recommendation regarding the availability of skilled personnel for provision of ENC to every baby after birth.^7^

Newborn deaths associated with conditions that are linked to poor quality of care during delivery can be easily prevented with proven cost-effective interventions.^8^

The purpose of the package for essential newborn care is to prevent and reduce neonatal mortality and morbidity.^3^ Factors that associated with essential new-born care for nurses and/or midwives have been associated with; lack of essential new-born care guidelines, lack of training related to ENC, shortage of staff in the respective units, lack of important equipment, and inadequate training and supervision.^9^

This study, therefore, aimed to assess essential new-born care knowledge and skills and associated factors among Nurses/Midwives in Zanzibar, Tanzania.

## METHODOLOGY

### Study Design and Setting

This study was a hospital-based analytical cross-sectional study involving quantitative approach. The study was conducted on 2 main islands; Pemba and Unguja in Zanzibar from January to February 2021. According to the National Census of 2012, Zanzibar had a total population of about 1,303,579, whereby Unguja was populated with a total of 898,721 people. In Zanzibar, healthcare service system is categorised into 3 levels: primary level comprises 122 Primary Health Care Units and Centres (PHCUs), among these, 53 are located in Pemba and 69 are located in Unguja, secondary level consists of 2 districts' hospitals situated in Pemba and one regional hospital situated in Unguja and tertiary level includes 2 specialised Hospitals located in Unguja. All health facilities mentioned above provide maternal and neonatal health services except for 40 PHCUs. The number of newborn birth in Zanzibar was estimated to be 41,639 in 2018.^10^ The neonatal mortality rate was 28 deaths per 1000 live births in 2015.^11^ Zanzibar was selected for this study because it has a high prevalence of neonatal mortality rate.

### Study Population

This study involved 246 nurses/midwives working in the delivery rooms, neonatal and premature units in selected public health facilities in Zanzibar. We included nurses/ midwives who were employed for at least one year before the time of data collection.

### Inclusion/ Exclusion Criteria

Only nurses/ midwives working in selected health facilities and were willing to participate in the study were included. All study participants consented to participating in this study. Nurse/midwives who did not consent were excluded.

### Sample Size and Sampling Technique

### Sample Size Determination

Sample size was calculated by using the Kish Leslie formula for cross-sectional study as it was used in our previous study conducted in Northeast Ethiopia ^12^.

n = Z^2^ p(1-p)/ e^2^.

Where n = minimum required sample size, z = confidence level at 95% (standard value of 1.96), and p = proportion of the estimated ENC, and marginal error of 0.05. We assumed the proportion to be 17.7% as was reported in a previous study which was conducted in Tigray, Ethiopia on Knowledge and practice of immediate new-born care among midwives ^13^

Therefore, the sample size was 222 and by assuming a 20% non-response rate, the final sample size (i.e.246*(1/1 − 0.10)) turned to be 267.

### Sampling Technique

Tertiary and regional hospitals were purposively selected with reason: having high number of new-borns. Simple random sampling method using the lottery replacement method was used to select 2 district hospitals, 4 cottage hospitals, and 31 Primary Health Care Centres (PHCC). Records of nurse and/ or midwives in all the 37 selected health facilities were reviewed. A total of 1,548 nurse and/ or midwives at all selected health facilities was observed. Proportionate Sampling technique was used to obtain the required number of nurses and/or midwives from each of the selected hospital using formula; ni = (Ni/ Nt) × n as used in a previous study.^12^ Where ni = required number of study participants from a given hospital, Ni = required sample size for the study, Nt = total number of nurses and/ or midwives from all selected hospitals, and n = number of nurses and/ or midwives in each of the selected health facility. The total number of nurse-midwives in the selected health facilities (n=1,548) and the sample size of 271 was proportionately allocated to the 37 health facilities. Study participants were selected using systematic random sampling technique in each given hospital. Nurses/Midwives who were able to respond to questions were approached after working hours.

### Data Collection Methods and Tools

A self-administered questionnaire was used to collect data on; knowledge, and reported skills of nurse-midwives on immediate new-born care and observational checklist for facility infrastructure, equipment, supplies and drugs used for ENC. The questionnaire was adopted from a previous study which was conducted in Ethiopia.^9^practice and associated factors among healthcare providers in Northwestern Zonal health facilities Tigray, Ethiopia, 2018. Results: Among the total healthcare providers, who participated in this study, 64.8% had good knowledge and 59.8% of the respondents had a good level of essential newborn care practice. Unavailability of adequate materials (like guidelines, drug, etc. The questionnaire was in line with the WHO guidelines for essential newborn care.^13^ The questionnaire was modified to fit the study's local context and purpose of the study. The tool was pretested on 27 (10%) of the total respondents. Nurse-midwives included in the pre-test were not included in the study. Following the pre-test, corrections to items that were not clear were made. The internal consistency of the tool for data collection was determined using Cronbach's alpha test, where alpha was found to be 0.87 for the tool assessing nurse-midwives knowledge on ENC. The questionnaire was organised into 3 parts: Part 1, was about the socio-demographic characteristic of nurse/midwives, part 2 included 6 questions for assessing nurses/midwives' knowledge of ENC, and part 3 contained 9 questions, which assessed the reported skills using 4 points Likert scale.^13^ The checklist was used for; recording drugs, supplies, equipment, and infrastructure which are used in ENC and was adopted from a previous study that was conducted in Ethiopia.^7^ The labour rooms, Operation Theatres (OTs), and Paediatric wards were examined for presence of New-born Care Corners (NCC). The NCC is defined as a special space within the labour room, OT and paediatric wards specifically for providing immediate newborn care to all new-borns.^14^ NCC area is equipped with radiant warmer to maintain neonate's temperature, also equipped with a resuscitation-kit for reviving asphyxiated neonates. Four research assistants (qualified registered nurses) were trained on how to collect data using the questionnaire and the observational checklist. Also, they were trained on ethical issues including confidentially.

### Variable and Measurement

Knowledge related to ENC was measured in response to 6 multiple choice questions. Those who scored above or equal to mean were considered as having adequate knowledge and those who scored below mean were considered as having inadequate knowledge. Reported skill regarding ENC was having 9 items and it was measured in response to 4 points Likert scale: “4” Consistently, “3” Regularly, “2” Rarely, and “1” Never.^15^ The nurse/midwives were asked to state how they perform ENC using the following clinical skills; observing the newborn at birth, drying the baby with a dry towel immediately after birth, and stimulate the baby while drying, assessing breathing and colour, early skin-to-skin placement, assessing eyes and apply tetracycline, examination of a newborn after delivery and before discharge, give vitamin K injection intramuscular on anterior mid-thigh and observation at the site of injection. Interpretation was as follows: good practice: if the nurse-midwives responded positive to more than or equal to 70% the practice procedures. Poor practice: if the nurse-midwives responded positive to less than 70% of the practice procedures.^16^

### Data Analysis

Statistical Package for a Social Sciences (SPSS v. 20) software program was used for data entry, processing, and analysis. Descriptive statistics were used to analyse the demographic characteristics of the respondent and results were presented in proportions. Pearson Chi-square statistical test was used to assess the association between categorical variables. To account for possible confounder while assessing the factors associated with ENC knowledge and skills, multiple logistic regression models were employed. The models included several variables reported to be associated with ENC knowledge and skills.^16–18^ A *p-value* of less than .05 was considered significant.

### Ethical Approval and Consent to Participate

Ethical approval was obtained from research and publication committee of the Dodoma University with approval no. Ref. MA.84/261/01/94. Verbal consent was requested from the participants after explaining the purpose of the study. A request for signing the written informed consent was made after the participants agreed to participate in the study. Participants were involved in this study voluntarily and were allowed to withdraw from the study at their convenience. In order to protect autonomy, privacy and confidentiality of participants, we used codes instead of actual names of the study participants and only principal researchers and the assistants were having access to the filled questionnaires.

## RESULTS

A total of 246 nurses/midwives participated in this study accounting to a 92.1% response rate. Participants were aged between 24 to 53 years with a mean age of 33.11 ± 5.96 years. The majority of the participants 232 (94.3%) were female. Most of them 147 (59.8%) were residing in Unguja. The majority of participants 174 (70.7%) had Ordinary Education Level. Regarding qualification, the majority 226 (91.9%) of them had a diploma in nursing. Most of the participants 77 (31.3%) had working experience of 3 to 4 years. Regarding health facility level, majority of the participants 180 (73.2%) were working in primary health care plus (Table 1).

**TABLE 1:** Demographic Characteristics of Respondents (N=246)

Variable	Frequency (n)	Percent (%)
Age (years)		
24–30	107	43.5
31–40	107	43.5
41 and above	32	13.0
Sex		
Male	14	5.7
Female	232	94.3
Residence		
Unguja	147	59.8
Pemba	99	40.2
Educational level		
O level	174	70.7
A level	72	29.3
Personal qualification		
Diploma of Nursing	226	91.9
Bachelor of Nursing	20	8.1
Working experience (years)		
1–3	69	28.1
4–6	77	31.3
7–9	55	22.4
10+	45	18.3
Health facility level		
Hospital	66	26.8
Primary Health care +	180	73.2
In service training		
Yes	82	33.3
No	164	66.7
Number of in-service trainings		
One to two	52	21.1
Three and above	30	12.2

### Availability of Essential Guideline, Equipment, Supplies and Drugs at the Point of Care

Out of the 38 health facilities visited, only 9 (23.7%) had guidelines for ENC. The assessment also observed critical shortage of ENC supplies and drug, majority of hospitals had inadequate supplies and drugs (Table 2)

**TABLE 2:** Availability of Essential Guidelines, Equipment, Supplies, and Drugs at the Point of Care

Guideline, equipment, supplies, and drugs	Health facilities		
Guideline	District Hospital n =3	Cottage hospitals n = 4	Primary health care centers (PHCC) n =31
Guidelines or protocol for essential newborn care	1 (33.3%)	2 (50.0%)	6 (19.4%)
Supplies and equipment			
Sterile scissors or blade	2 (66.7%)	1 (25.0%)	10 (32.0%)
Sterile disposable cord ties or clamps	3 (100.0%)	3 (75.0%)	10 (32.0%)
Towel or blanket to wrap baby	1 (33.3%)	1 (25.0%)	0 (0.0%)
Functional ambu bag (250 or 500mL self-inflating bag)	1 (33.3%)	1 (25.0%)	5 (16.1%)
Functional mask size 0 (preterm and low-birth-weight baby)	1 (33.3%)	1 (25.0%)	5 (16.1%)
Functional mask size 1 (term baby)	1 (33.3%)	1 (25.0%)	5 (16.1%)
Drugs			
Tetracycline ointment	2 (66.7%)	3 (75.0%)	11 (35.5%)
Injectable vitamin K	1 (33.3%)	1 (25.0)	4 (12.9%)

### Knowledge and Skills of Nurses/Midwives Regarding Essential New-born Care

The majority 89 (36.2%) and 66 (27%) of participants had adequate knowledge and appropriate skills regarding essential newborn care, respectively (Figure 1).

### Association between Knowledge and Socio-demographic Characteristics among Nurses-Midwives towards Essential New-born Care

Univariate results indicated that, knowledge of essential newborn care was significantly associated with age, residence, professional qualification, health facility level, work experience, ENC training, availability of ENC guidelines, availability of drugs and supervision. After controlling for confounders, nurse/midwives' knowledge was significantly associated with professional qualification and availability of ENC guidelines. Respondents with a Bachelor of Science in Nursing were significantly (8.8 times) more likely to have adequate knowledge compared to those with a diploma in Nursing (AOR=8.83, *p=.0040*). Those nurses/ midwives who had ENC guidelines were significantly more likely to have adequate knowledge compared to those who had no guidelines in their facilities (AOR=3.52, *p=.0020*). Other factors, like supervision, shortage of staff, availability of drugs, ENC training, and demographic characteristics were not significantly associated with knowledge (Table 3).

**TABLE 3:** Factors Associated with Knowledge and Socio-demographic Characteristics among Nurses/ Midwives (N=246)

Variable	Unadjusted analysis		Adjusted analysis	
	OR [95%CI]	p-value	AOR [95%CI]	p-value
Age (years)				
24-30	Reference			
31-40	2.92 [1.57, 5.44]	.0007	1.53 [0.42, 5.51]	.5164
41+	15.54 [5.90, 40.94]	<.0001	6.41 [0.84, 48.74]	.0728
Resident				
Unguja	Reference			
Pemba	0.57 [0.33, 0.99]	.0453	0.45 [020, 1.01]	.052
Educational level				
O-level	Reference			
A-level	0.36 [0.19, 0.69]	.0021	0.55 [0.21, 1.46]	.2298
Profession qualification				
Diploma of Nursing	Reference			
Bachelor of Nursing	12.37 [3.51, 43.57]	<.0001	8.83 [2.00, 38.96]	.004
Health facility level				
Hospital	3.53 [1.96, 6.35]	<.0001	1.59 [0.43, 5.95]	.4886
Primary Health Care plus	Reference			
Work experience (years)				
1-3	Reference			
4–6	0.87 [0.38, 1.99]	.7468	0.560.17, 1.89]	.353
7–9	4.07 [1.85, 8.98]	.0005	2.25 [0.51, 9.98]	.2874
10+	9.67 [4.05, 23.12]	<.0001	2.30 [0.34, 15.82]	.3964
Training ENC				
Yes	4.03 [2.30, 7.07]	<.0001	1.75 [0.72, 4.26]	.2205
No	Reference			
Present guideline				
Yes	3.23 [1.86, 5.60]	<.0001	3.52 [1.59, 7.80]	.002
No	Reference			
Availability of drugs				
Yes	3.75 [1.98, 7.13]	<.0001	2.28 [0.87, 5.95]	.0927
No	Reference			
Shortage of staffs				
Yes	0.47 [0.21, 1.09]	.079	0.87 [0.28, 2.75]	.8129
No	2.11 [0.92, 4.85]			
Supervision				
Yes	3.43 [1.65, 7.12]	.0009	0.79 [0.26, 2.44]	.6822
No	Reference			

### The Reported Skills of Essential New-born Care

The majority of respondents, 166 (67.5%), stated that they care for newborns immediately after delivery; of them, 106 (43.1%) agreed to dry babies with dry towels immediately after birth. 10 (4.1%) strongly agreed that they assess newborn's respiration and color. 14 (5.7%) of them outright agreed to cut umbilical cords between 1 and 3 centimeters. Only 10 (4.1%) nurse-midwives strongly agree to apply tetracycline and care for the newborn's eyes. Most participants 63 (25.6%) strongly agreed to conduct a physical examination of the newborns immediately after birth and before discharge. Most of the participants, 63 (25.6%) strongly agreed to place the newborns skin-to-skin contact with their mothers and early initiation of breastfeeding immediately after the baby is born (Table 4).

**TABLE 4:** Reported Skills Regarding Nurses/Midwives on Essential Newborn Care

Variable	Never=1 n (%)	Rarely=2 n (%)	Regularly=3	Consistently=4 n (%)	Mean (SD)
Observing newborn at birth	48 (19.51)	21 (8.54)	166 (67.48)	11 (4.47)	2.68 (0.69)
Immediately after birth dry the baby with a dry towel	42 (17.07)	22 (8.94)	106 (43.09)	76 (30.89)	2.96 (0.92)
Assessing the breathing and color of the newborn,	92 (37.40)	56 (22.76)	88 (35.77)	10 (4.07)	2.21 (0.84)
Cutting cord 1_3cm	122 (49.59)	67 (27.24)	43 (17.48)	14 (5.69)	2.02 (0.82)
Assessing eyes and applying tetracycline	126 (51.22)	52 (21.14)	58 (23.58)	10 (4.07)	2.11 (0.76)
Place the baby skin-to-skin contact and early initiation of breastfeeding	38 (15.45)	30 (12.20)	115 (46.75)	63 (25.61)	2.86 (0.94)
Give vitamin K injection IM on anterior mid-thigh	112 (45.53)	109 (44.31)	22 (8.94)	3 (1.22)	1.67 (0.69)
observing the site of injection.	116 (47.15)	57 (23.17)	63 (25.61)	10 (4.07)	2.11 (0.80)
Examination of a newborn after delivery and before discharge	116 (47.15)	91 (36.99)	29 (11.79)	10 (4.07)	1.83 (0.79)
Overall skills	180 (73.17)	0 (0.00)	66 (26.83)	0 (0.00)	2.27 (0.44)

### Factors Associated with Skills Regarding Nurses/ Midwives on Essential Newborn Care

After controlling for confounders, nurse/midwives residing in Pemba were significant less likely to have appropriate skills as compared to those residing in Unguja (AOR=0.30, *p=.0242*). With regard to shortage of staff, it was noted that respondents who reported to have shortage of staffs were significant less likely to have appropriate skills as compared to those with enough staff (AOR=0.08, *p=.0003*). Respondents who had knowledge on essential newborn care were almost 3 times more likely to have appropriate skills compared to respondents with inadequate knowledge (AOR=2.80, *p=.0235*) (Table 5).

**TABLE 5:** Association between Skills, Knowledge, and Social-demographic characteristics among Nurses/Midwives toward ENC (N=246)

Variable	Unadjusted analysis		Adjusted analysis	
	OR [95%CI]	p-value	OR [95%CI]	p-value
Age (years)				
24–30	reference			
31–40	1.54 [0.81, 2.95]	0.1915	0.98 [0.21, 4.64]	0.9833
41+	5.59 [2.39, 13.10]	<0.0001	0.50 [0.05, 5.20]	0.5644
Resident				
Unguja	Reference			
Pemba	0.30 [0.16, 0.58]	0.0004	0.30 [0.11, 0.86]	0.0242
Educational level				
O level	Reference			
A level	0.57 [0.29, 1.10]	0.0952	0.85 [0.30, 2.47]	0.7698
Personal qualification				
Diploma of Nursing	reference			
Bachelor of Nursing	3.80 [1.50, 9.66]	0.0050	1.64 [0.48, 5.62]	0.4307
Health facility level				
Hospital	1.88 [1.02, 3.45]	0.0425	1,41 [0.33, 5.95]	0.6429
Primary Health care +	Reference			
Working experience (years)				
1–3	reference			
4–6	0.60 [0.26, 1.41]	0.2428	0.92 [0.21, 3.96]	0.9077
7–9	1.35 [0.59, 3.08]	0.4755	1.11 [0.20, 6.33]	0.9053
10+	4.50 [1.98, 10.22]	0.0003	4.89 [0.54, 44.10]	0.1570
Training ENC				
Yes	2.45 [1.37, 4.38]	0.0026	1.59 [0.62, 4.13]	0.3371
No	Reference			
Present guideline				
Yes	2.80 [1.54, 5.09]	.0007	1.51 [0.61, 3.77]	.3748
No	Reference			
Availability of equipment				
Yes	0.52 [0.29, 0.92]	.0242	0.64 [0.23, 1.79]	.3978
No	Reference			
Availability of drugs				
Yes	2.60 [1.36, 4.98]	.0038	2.15 [0.73, 6.38]	.1661
No	reference			
Shortage of staffs				
Yes	0.07 [0.023, 0.185]	<.0001	0.08 [0.02, 0.32]	.0003
No	Reference			
Supervision				
Yes	2.56 [1.23, 5.31]	.0116	0.77 [0.24, 2.47]	.6574
No	Reference			
Knowledge				
Adequate	5.60 [3.05, 10.31]	<.0001	2.80 [1.15, 6.84]	.0235
In adequate	Reference			

## DISCUSSION

Essential newborn care is of paramount important for the health of the newborn and its survival. Midwives' adequate knowledge and appropriate skills on ENC at time of delivery and afterwards determine the newborn's health outcome.^14^ Additionally, research shows that effective skills of ENC avert about 50% to 75% of newborn deaths during delivery and postnatal period respectively^5^, as this highly depends on the competence of nurse/midwives on how they provide ENC. Key findings in this study include positive association between professional qualification, availability of guideline and nurse/midwives' adequate knowledge on ENC. On the other hand, ENC skills were positively associated with midwives residing in Pemba (urban), availability of staff and adequate knowledge.

In the current study, only 36.2% of midwives had adequate knowledge on ENC. This result is more or less similar to results observed in a study conducted in Uganda which reported ENC among midwives to be at 46.5%.^19^ Contrary to our findings, a study conducted in Ethiopia reported that 74.7% of the midwives had adequate knowledge on newborn care ^18^504, out of this 84,437 was from neonatal death and this mortality is related to immediate obstetric and newborn care of babies provided by health care providers; But little was known about the level of knowledge and practice related to immediate newborn care and their associated factors among health care providers generally in Tigray region and specifically in the Eastern Zone so the aim of this study was to assess knowledge and practice of immediate newborn care and associated factors among health care providers in the Eastern zone public health facilities, Tigray, Ethiopia. Methods: A cross-sectional study was conducted from December 2015 to February 2016. A total of 16 health care facilities were selected for study using simple random sampling techniques and all health care providers in the selected health care facilities who participated in immediate newborn care were involved in the study. Data were entered, cleaned and analyzed using SPSS version 20.0. Ethical clearance was obtained from Adigrat University institutional ethical review board and Tigray regional health bureau. Consent was obtained from participants to conduct the study. Result: In this study 215 participants were contacted, with a response rate of 99.1%. Generally, from the health care providers who participated in this study, 74.65% had adequate knowledge on newborn care and overall 72.77% of the participants were having good newborn care practice. Among the health care providers participated in the study, 151 (70.9%. Another study conducted in Afghanistan by reported that 66% of midwives had adequate knowledge on ENC.^20^

Baseline findings from study conducted in Zambia also reported contrary findings of nurses' knowledge (65%) on ENC.^21^ This study's finding is smaller compared to findings of a study done in Ethiopia (57.9%).^7^ The difference observed might be due to the in-service training regarding ENC and professional qualification. In our study, midwives who received in-service training on ENC and diploma in nursing were only 33.3% and 91.9%, respectively. Study done in Northwest Ethiopia and eastern Tigray reported that obstetric health care providers who received in-service training were 45.3% and approximately 70%, respectively. Moreover, participants in the study conducted in Northwest Ethiopia and eastern Tigray who had diploma in nursing were 46.8% and 69.6%, respectively.^14^

Regarding midwives' skills for ENC, only 27% of them had appropriate skills. Our finding was contrary to study conducted in Ethiopia by Asteray et al which reported that 62.7% of obstetric care providers had appropriate ENC skills.^19^ Another study conducted in Tigray, Ethiopia in 2016 reported that, 72.8% of the study participants had appropriate ENC skills.^16^ The difference observed in our study and other studies' findings may be associated with low level of professional qualification as majority (91.9%) of nurse and / or midwives in our study were having a diploma in nursing and with inadequate in-service training. It is reported that regularly in-service training regarding ENC improves daily hands-on skills.^16^

In the current study, nurse-midwives who had a bachelor degree were almost 9 times more likely to have adequate knowledge on ENC compared to those with diploma level of education. This keeps in line with findings reported elsewhere.^22, 23^ The similarity in the findings may be due to the training curriculum and the duration of training. In our study's area, training for diploma in nursing is for 3 years without internship meanwhile bachelors of science in nursing is for 4 years plus an additional 1 year for internship. Thus, nurses with bachelors of science in nursing have more exposure in neonatal care compared to those with a diploma in nursing.

Availability of guideline on ENC in this study was positively associated with adequate knowledge. This observation is similar to results of a study conducted in Tigray, Ethiopia which reported that, availability of materials including guidelines for ENC were significantly associated with adequate knowledge and practice of ENC.^9^

Working experience of 7 years and above was a predictor for nurses-midwives' knowledge on ENC. However, it was not statistically significant. This keeps in line with the findings reported in a study conducted in Tanzania which reports that nurses who had 5 years and above working experience had adequate knowledge on newborn resuscitation.^18^

This study also assessed midwives' skills on essential newborn care. Eight important components of ENC were assessed. These include; stimulation of baby to breath, assessment of breathing, the newborn being kept warm immediately after birth, cord-care, initiating breastfeeding within the 1st hour of delivery, administration of eye ointment, and administration of vitamin K.^23^ Majority (95%) of the participants claimed that they comply with ENC guidelines. However, when observed while conducting deliveries, they had poor skills relating to ENC. For example, only 39. 84% assessed breathing and the colour of the newborn, 27.65% of the midwives assessed the eyes of the newborn and applied tetracycline; 15.86% examined the newborn after delivery, 10.16% placed the baby's skin to skin contact of the mom's abdomen and ensured initial breastfeeding; 29.68% gave injection of Vitamin K, and only 26.83% observed infections of the code.

Indeed, the nurse-midwives' skills are poor to an alarming situation. This observation is backed-up by finding from other studies which reports that the knowledge of nurses/midwives is inadequate.^25,26^ This situation poses threats to not only the health of the newborn baby, but also to the health of their mothers. It doesn't appeal into the intuition that a nurse who performs poorly to newborns would, for example, do better in taking care of a Caesarean section wound. The poor skills observed through this research could explain why 99% of the deaths occur in low-income nations and occur, particularly in the early stages after birth.^1^, Zanzibar being among the countries with high mortality rates. While low knowledge of ENC among nurses and/or midwives could be the reason for the poor skills, lack of staff nurses-midwives and poor facilities form another reason for the poor skills. It is argued that there are poor quality facilities in the third world countries and thus the increasing number of newborn death.^24^ Another cause of inappropriate ENC skills may be due to unavailability of guideline for ENC in the labour wards.

Nurse/midwives who were having adequate knowledge on ENC were almost 3-fold more likely to have appropriate skills. This keeps in line with findings of a study conducted elsewhere which reported that high odds of inadequate knowledge influence poor practice of ENC.^25^ In another study conducted in Ethiopia, adequate knowledge was found to be significantly associated with good practice on ENC.^26^ A similar study conducted among Health Professionals in Governmental Health Facilities of Bahir Dar City and Gulomekada District also reported that good knowledge regarding ENC influence appropriate practices.^27^

Regarding shortage of staff, the current study found that health facilities with shortage of staff were less likely (AOR = 0.08) to have appropriate skills related to ENC. Similar findings were reported in a study conducted in Northeast Ethiopia.^25^ Research shows that effective skills of ENC avert about 50% to 75% of newborn deaths during delivery and postnatal period respectively^5^, and this is highly dependent on the competence of nurse/midwives.

## CONCLUSION AND RECOMMENDATION

Generally, nurses-midwives had suboptimal knowledge and skills on essential newborn care. In order to rescue the situation, Nurses-midwives are in urgent need of positive supportive supervision and low dose–high frequency skills training in ENC for prevention of neonatal morbidity and mortality. Also, policymakers should be aware of this gap and should plan necessary interventions to close the gap.

### Limitation

Self-reported practice has recall bias, also a cross sectional study design does not show temporal cause and effects. Future studies should address these limitations.
